# Identification and Verification of m^7^G Modification Patterns and Characterization of Tumor Microenvironment Infiltration *via* Multi-Omics Analysis in Clear Cell Renal Cell Carcinoma

**DOI:** 10.3389/fimmu.2022.874792

**Published:** 2022-05-03

**Authors:** Kai Dong, Di Gu, Jiazi Shi, Yewei Bao, Zhibin Fu, Yu Fang, Le Qu, Wentong Zhu, Aimin Jiang, Linhui Wang

**Affiliations:** ^1^ Department of Urology, Changhai Hospital, Naval Medical University, Shanghai, China; ^2^ Department of Urology, Changzheng Hospital, Naval Medical University, Shanghai, China; ^3^ Department of Urology, Affiliated Jinling Hospital, Medical School of Nanjing University, Nanjing, China; ^4^ School of Chinese Medicine, Chinese University of Hong Kong, Shatin, Hong Kong SAR, China

**Keywords:** N7-methylguanosine, immune microenvironment, single cell, prognosis, drug response, renal cell carcinoma

## Abstract

The epigenetic modification of tumorigenesis and progression in neoplasm has been demonstrated in recent studies. Nevertheless, the underlying association of N7-methylguanosine (m^7^G) regulation with molecular heterogeneity and tumor microenvironment (TME) in clear cell renal cell carcinoma (ccRCC) remains unknown. We explored the expression profiles and genetic variation features of m^7^G regulators and identified their correlations with patient outcomes in pan-cancer. Three distinct m^7^G modification patterns, including MGCS1, MGCS2, and MGCS3, were further determined and systematically characterized *via* multi-omics data in ccRCC. Compared with the other two subtypes, patients in MGCS3 exhibited a lower clinical stage/grade and better prognosis. MGCS1 showed the lowest enrichment of metabolic activities. MGCS2 was characterized by the suppression of immunity. We then established and validated a scoring tool named m7Sig, which could predict the prognosis of ccRCC patients. This study revealed that m^7^G modification played a vital role in the formation of the tumor microenvironment in ccRCC. Evaluating the m^7^G modification landscape helps us to raise awareness and strengthen the understanding of ccRCC’s characterization and, furthermore, to guide future clinical decision making.

## Introduction

Renal cell carcinoma (RCC) is one of the 10 most prevalent cancers worldwide (1), and it is estimated that there are more than 430,000 incident RCC patients each year globally and of which approximately 180,000 deaths are reported (2). Clear cell renal cell carcinoma (ccRCC) is the most common histological type, comprising over 75% of all RCC cases (1), and it is characterized by invasive growth, high rates of metastasis, and poor outcomes (3). Besides, metastasis of ccRCC is the most dominant reason for cancer-related death and treatment failure (4). Although surgical excision produces favorable results to treat localized ccRCC, approximately one-third of patients will eventually develop tumor recurrence and progression after surgical resection of primary lesions (5, 6). In addition, ccRCC is not sensitive to radiotherapy and chemotherapy. Although targeted therapy and immunotherapy achieve effect in the treatment of ccRCC, many patients have intrinsic resistance or will eventually develop acquired resistance (7, 8). Unfortunately, the 5-year survival of patients with advanced ccRCC is less than 10% (1). In current practice, the most used models for risk stratification and prognostic prediction are Fuhrman nuclear grade and TNM classification system (9). Owing to the intra‐tumor heterogeneity, patients with similar clinical characteristics may have considerably different prognoses (10). Tumor heterogeneity could also contribute to drug resistance and metastasis (11). Therefore, there is still an urgent need to mine the prognostic markers and fully elucidate the molecular mechanism associated with the tumorigenesis and progression of ccRCC.

The epigenetic modification of RNA has received extensive attention owing to its vital role in the regulation of diverse biological activities (12). In eukaryotic cells, more than 170 types of post‐transcriptional RNA modifications have been identified (13). As one of the most common modifications, N7-methylguanosine (m7G) occurs in transfer RNA (tRNA) (14), microRNA (15), ribosomal RNA (rRNA) (16), the 5′cap (17), and internal regions (18) of mRNA. It is reported that the disorder of WDR4/METTL1‐mediated m^7^G modification was correlated with primordial dwarfism (PD) (19). Recently there has been growing interest in finding out what role the m^7^G modification exactly plays in cancer. METTL1-mediated m^7^G tRNA modification could promote the progression of intrahepatic cholangiocarcinoma (20), lung cancer (21), and bladder cancer (22). However, the function of m^7^G in the tumorigenesis and progression of ccRCC remains unknown.

In this study, we performed m^7^G -related gene signature research by pan-cancer analysis, then identified molecular features, biological function, tumor microenvironment infiltration, and clinical relevance of distinct m^7^G modification patterns in ccRCC by integrating multi-omics data. A scoring tool, named m7Sig, was also constructed and verified to predict the outcome of patients with ccRCC.

## Materials and Methods

### Patient and Clinical Samples

Overall, 50 pairs of ccRCC and adjacent non-cancerous tissues and a cohort of 70 ccRCC tissues were collected from Changzheng Hospital (Shanghai, China). All samples were reviewed by two pathologists. All patients provided informed consent, and the protocol of this study was approved by the Ethics Committee of Changzheng Hospital.

### RNA Isolation and RT-qPCR

Total RNA was extracted by Trizol (Invitrogen, USA) and then reverse-transcribed using commercial kits (Takara, Japan). RT-qPCR was performed with a LightCycler 480 (Roche, Germany) and relative expression levels of EIF4A1 were normalized to GAPDH using 2–ΔΔCT method. Primer sequences for EIF4A1 (Forward: AAGCCGTGGATTCAAGGACCAG, Reverse: CACCTCAAGCACATCAGAAGGC); Primer sequences for GAPDH (Forward: GTCTCCTCTGACTTCAACAGCG, Reverse: ACCACCCTGTTGCTGTAGCCAA).

### Immunohistochemistry

Immunohistochemical (IHC) staining was performed with EIF4A1 antibody (ab31217, Abcam) following the previous protocol (23). The IHC scores were calculated by staining intensity and the percentage of stained cells as reported (24).

### Data Collection and Processing

Normalized expression data, DNA methylation, TMB, and clinical data in The Cancer Genome Atlas (TCGA) were obtained from UCSC Xena datasets (including ccRCC cohort) (25). CNV and somatic mutation data of ccRCC were derived from the GDC portal. Out-house datasets, including gene expression and clinical information of the Japan renal cancer cohort, were downloaded from phs002252.v1.p1 (26). The ccRCC single-cell RNA-sequence data of PRJNA705464 were obtained from the GEO database (27). Multiple public databases, including UALCAN, TIMER, TIDE, and MEXPRESS, were also acquired in this study. For datasets in public datasets, informed consent and instructional review board approval were not required.

### Identification of Different m^7^G Subgroups in ccRCC

Altogether, we collected 28 7-Methygunaosime (m^7^G) modification-related genes from prior articles, reviews, and databases (Reactome, CPDB, KEGG, MSigDB) (28–36) (**Table S1**). The Spearman’s and Pearson’s rank correlations between m^7^G genes were assessed with the R package “corrplot”. Consensus clustering was conducted according to the expression profile of the m^7^G related genes using the R package “ConsensusClusterPlus.” (Detailed parameters: reps=100, pItem=0.8, clusterAlg=“km”, distance=“euclidean”). Then, 531 ccRCC patients were grouped into distinct subtypes using PCA *via* R package “ConsensusClusterPlus”, and k = 3 was identified as the best subtype number.

### Enrichment Analysis Among Subgroups

R package “DEseq2” was utilized to identify different expression genes (DEGs) among subgroups, threshold values were set with p-adjusted value < 0.01, and abstract log-fold change = 2. The R package “ClusterProfiler” was applied to perform Kyoto Encyclopedia of Genes and Genomes (KEGG) pathway and Gene set variation analysis (GSVA). All the gmt files for enrichment analysis were obtained from the MSigDB database (28) and the ConsensusPathDB database (30).

### Analysis of Tumor Microenvironment (TME)

Several immune cell infiltration algorithms, including TIMER, CIBERSORT, QUANTISEQ, MCPCOUNTER, XCELL, and EPIC, were used to compare the immune landscape among subgroups. In addition, single sample gene set enrichment analysis (ssGSVA) was employed to further validate the immune cell infiltration difference in ccRCC subgroups (37–40). The infiltration extent of immune and stromal score in ccRCC was calculated with R package “ESTIMATE”. Tumor Immune Dysfunction and Exclusion (TIDE) algorithms (41) were utilized to compare the immune therapy response among different subgroups.

### Mutation Profile Among Subgroups

R package “Maftools”, famous for its convenience to analyze the somatic data and visualize, was utilized to compare the different mutation patterns among subgroups (42). Aided by the function of correlation function from “Maftools”, we calculated the mutation profile in distinct m^7^G subgroups as previously reported (43). Drug-gene interactions and oncogenic pathways were analyzed through the alteration analysis function module. Recurrent broad and focal somatic copy-number alteration (SCNA) analysis was performed by the GISTIC 2.0 (44).

### Assessment of Difference in Chemotherapy Response Among Subgroups

R “pRRophetic” package was used to assess the half-maximal inhibitory concentration. The difference in response to chemotherapy molecules and small pre-clinical drugs among subgroups was analyzed *via* public pharmacogenomics database (Genomics of Drug Sensitivity in Cancer, GDSC) (45). In addition, CellMiner database (46) and CCLE database (47) were introduced to compare the different sensitivity among ccRCC cell lines (48).

### Single-Cell Analysis

David et al. provided a large ccRCC single-cell cohort dataset, which consisted of different stage renal cancer tissue and normal tissue and a total of 164,722 single cells. We used this dataset to explore the role of m^7^G genes at the single-cell level. R package “Seurat” was used to perform dimension reduction and clustering analysis, and the annotation of cell cluster was obtained by R package “SingleR” (49).

### Construction and Validation of m^7^G -Related Risk Prognostic Signature

Using subgroup-related genes expression and overall survival data from the TCGA-ccRCC cohort, we firstly perform univariate COX regression to select survival-related genes. Then, the random survival forest variable hunting (RSFVH) algorithm was further utilized to determine the important signatures, which were used to establish a scoring tool (m7Sig): m7Sig risk score =β_1_xGene_1_ + β_2_xGene_2_ + β_3_xGene_3_ + ⋯β_n_xGene_n_. (N, the number of risk signatures; x, gene expression value; β, the coefficient of genes in the COX regression model). Japan cohort was utilized to validate our risk model and patients from those two datasets were classified into high- and low-risk subgroups based on the median m7Sig score.

### Statistical Analysis

Quantitative data obtained from experiments were presented as mean ± SD. Kruskal-Wallis test was applied to compare continuable variables among three groups. Student’s t-test and Wilcoxon test were introduced for two groups. Chi-squared test was utilized to identify the difference in classified variables including clinical characteristics among subgroups. Kaplan-Meier method and log-rank test were employed to assess the prognostic difference. All comparisons were two-sided. P-value < 0.05 was regarded as statistically significant. P values were indicated by * P < 0.05, ** P < 0.01, ***P < 0.001. Benjamini-Hochberg (BH) multiple test correction was used to calculate the adjusted P-value. R (version 4.0.4) and GraphPad Prism 7.0 were adopted for data processing, statistical analysis, and graphing.

## Results

### Disrupted m^7^G Regulators in Cancers and Their Correlations With Patient Outcomes

The study flow is shown in **Figure S1**. To globally understand the regulation pattern of m^7^G in cancer, we explored and verified the mRNA expression of m^7^G regulators in pan-cancer. The results showed m^7^G methyltransferases, such as METTL1 and WDR4, were upregulated in a wide range of cancers, while RNA-binding and decapping enzymes (NUDT4, NUDT16, and NUDT10) were significantly downregulated. (**Figure S2A**). We calculated the correlation of m^7^G-related genes in the TCGA-ccRCC expression matrix in two ways including Spearman (up-right) and Pearson (low-left) correlation test, results indicated that multiple genes had significantly correlative expression patterns (**Figure S2B**). Next, we determined the association between transcript levels and patient outcomes (**Figure S2C**), which indicated that the disturbed expression of m^7^G regulators exerted a non-negligible effect on cancer progression.

### Copy Number Variation and Sequence Mutation Lead to Dysregulated m^7^G-Regulator Levels in Cancers

**Figure 1:**
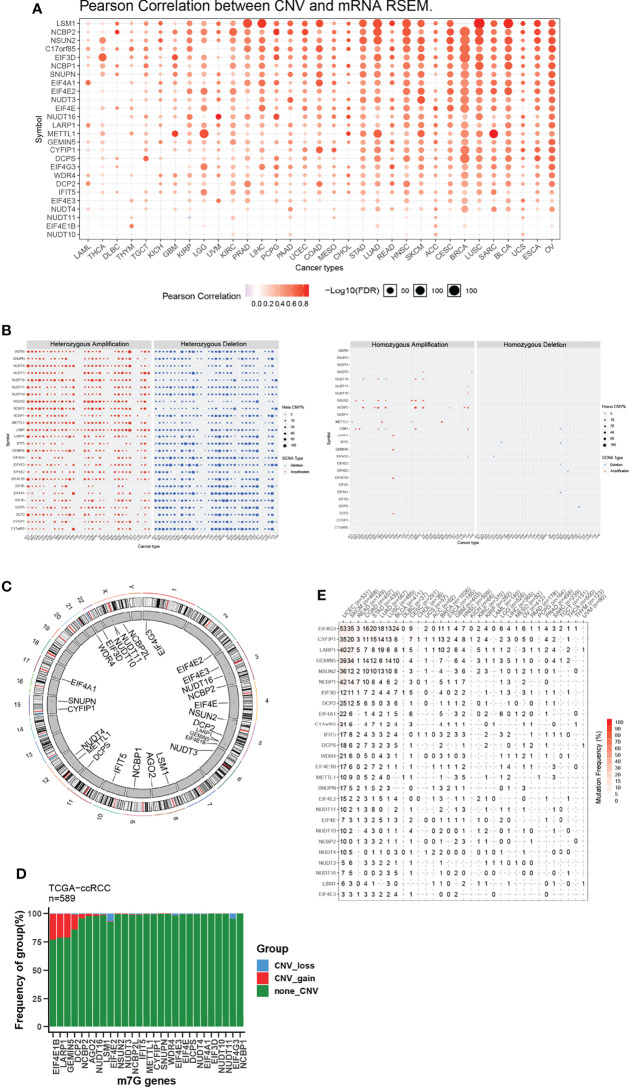
CNV and sequence alteration contribute to abnormal m^7^G-Regulator Levels. **(A)** CNV strongly correlates to gene expression of m^7^G regulators in pan-cancer using Pearson analysis. **(B)** Heterozygous and homozygous amplification/deletion of m^7^G regulators in pan-cancer. Amplification, red; Deletion, blue. **(C)** The location of CNV of m^7^G regulators on 23 chromosomes. **(D)** CNV of m^7^G regulators in TCGA-ccRCC dataset. CNV loss, blue; CNV gain, red; none CNV, green. **(E)** Mutation frequency of m^7^G regulators in pan-cancer.

To further explore why these m^7^G-related genes changed, we verified the copy number variation (CNV) in cancers and observed a clear positive correlation between CNV and mRNA expression (**Figure 1A**). As shown in **Figure 1B**, METTL1, NCBP2, and NSUN2 were frequently heterozygous amplified, but NUDT10 and EIF4E3 were dominantly heterozygous deletion. By contrast, homozygous amplification and deletion occurred at very low frequencies. The location of CNV alteration of m^7^G regulators on chromosomes is shown (**Figure 1C**). In ccRCC, we observed CNV gain for EIF4E1B, LARP1, GEMIN5, and DCP2, while EIF4E2 and EIF4G3 mainly had a frequency of CNV deletion (**Figure 1D**).

We also analyzed the mutation status of m^7^G genes and found the majority of them in certain tumor types, including UCEC, SKCM, COAD, STAD, LUAD, LUSC, and BLCA, were frequently mutated (**Figure 1E**). Our results demonstrated that both transcriptional dysregulation and DNA sequence alteration might influence m^7^G genes in cancer.

### Identification of Three Clusters by Consensus Clustering of m^7^G Regulators in ccRCC

**Figure 2:**
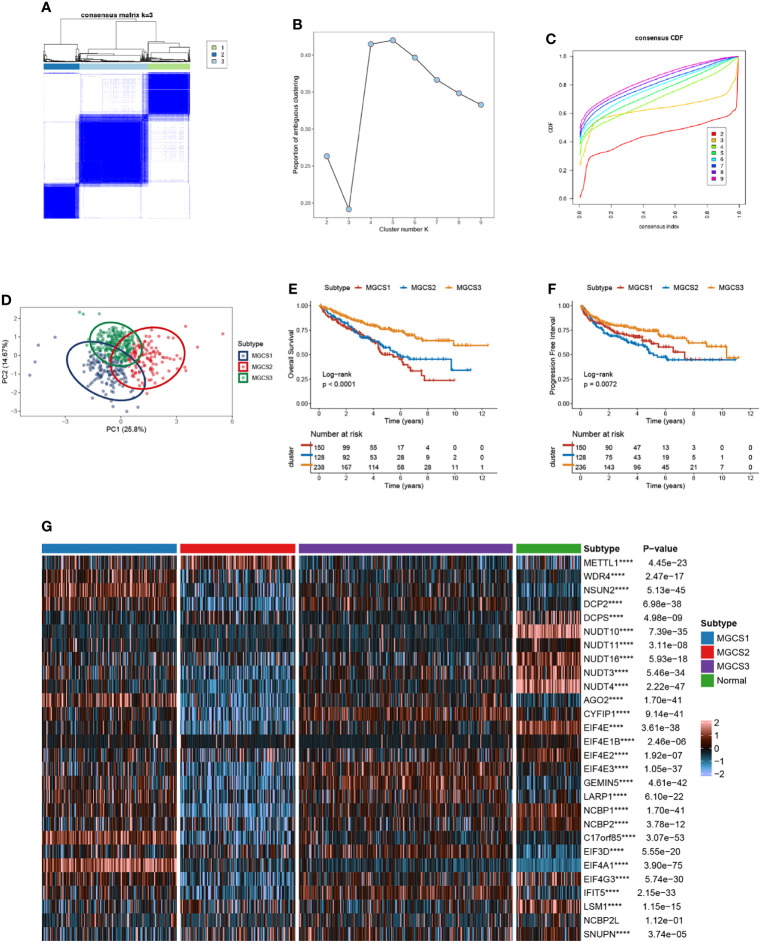
Identification of m^7^G subtypes of ccRCC. **(A)** Consensus matrix of samples in TCGA-ccRCC for k=3. **(B)** The number of optimum clusters is determined by the lowest proportion of ambiguous clustering. **(C)** The cumulative distribution function curves for k = 2 to 9. **(D)** The principal component plot is based on m^7^G -related genes. **(E, F)** Kaplan-Meier survival analysis for overall survival (left) and progression-free interval (right) of the three subtypes in TCGA-ccRCC dataset. **(G)** The expression profiles of the m^7^G regulators in three subtypes and normal kidney samples. ****P < 0.0001.

According to m^7^G -regulator levels, an unsupervised clustering method was used to group the TCGA-ccRCC samples into different molecular subtypes. As indicated in **Figures 2A–D**, we classified ccRCC into three distinct clusters, namely m^7^G -associated cancer subtype 1 (MGCS1), MGCS2, and MGCS3. The clinical significance of this typing method was assessed by comparison of clinicopathological features (**Table S2**) and clinical outcomes (**Figures 2E, F**) for the three main m^7^G modification subtypes. Compared with the other two subgroups, patients in MGCS3 exhibited a particularly prominent survival advantage. Moreover, we found that most m^7^G -related genes were significantly downregulated in MGCS2 (**Figure 2G**).

To further depict the features and potential structures of the distribution of every patient, we cast each patient into a manifold with sparse tree structures to confirm the risk landscape of ccRCC, as previously described (50). Consistently, patients with ccRCC were clearly separated into three clusters and showed distinct states (**Figures S3A, B**). Meanwhile, individual patient trajectory analysis and pseudotime ordering showed a risk transition trajectory (**Figure S3C**).

### Functional Enrichment Analysis in Distinct m^7^G Modification Patterns

**Figure 3:**
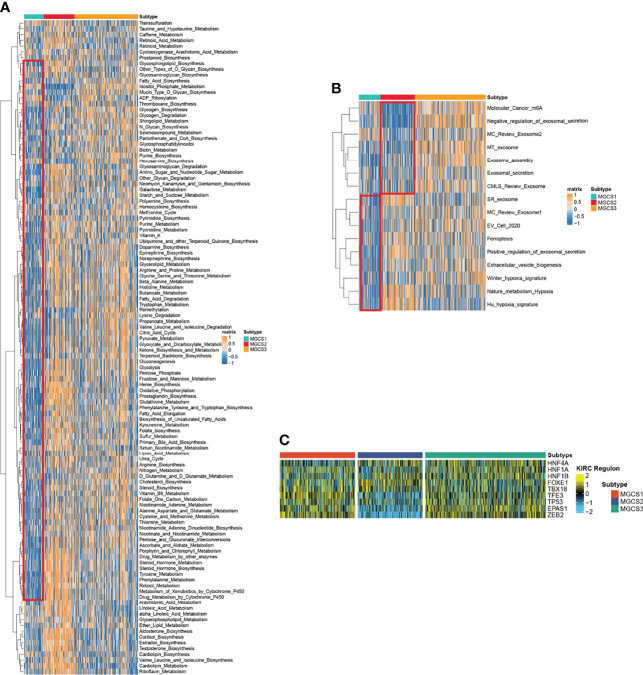
Functional enrichment analysis of MGCS1, MGCS2 and MGCS3 subgroups. **(A, B)** Heatmap of metabolism-related and cancer-related pathway enrichment scores among the subtypes by GSVA analysis. Orange represented activated pathways and blue represented inhibited pathways. **(C)** Heatmap of transcription factor regulon activation in three m^7^G modification subtypes. Yellow represented transcription factor regulation-activation. Blue represented transcription factor regulation-inhibition.

We then performed GSVA analysis regarding metabolism-associated signatures. Repression of metabolic status was observed in MGCS1, since multiple metabolic signatures including glycogen metabolism, purine metabolism, fatty acid degradation, pyruvate metabolism, glycolysis, gluconeogenesis, oxidative phosphorylation, glutathione metabolism, tyrosine metabolism, and retinol metabolism were suppressed in MGCS1. In contrast, most of these signatures were activated in MGCS2, suggesting a metabolically active state (**Figure 3A**). Consistently, the hypoxia-associated signature was enriched in MGCS2 through GSVA analysis (**Figure 3B**). Tumor hypoxia was reported to drive resistance to immunotherapy in cancer (51–53), so targeted hypoxia reduction may have the potential to sensitize MGCS2 to immunotherapy. In addition, m^6^A modification-related signature was inhibited obviously in MGCS2, indicating a potential connection between m^7^G and m^6^A (**Figure 3B**).

To further investigate the transcriptome differences, we analyzed regulons for m^7^G subtype-specific transcription factors from the obtained lists of renal cancer-associated transcription factors (26, 54, 55) using R package RTNduals (56), which rendered strong support to the biological pertinency of the three-classification because the regulon activity was closely related to m^7^G subtypes (**Figure 3C**). We also noted that ZEB2 exhibited the lowest activity in the MGCS2 group, suggesting the inhibition of the EMT process in this subtype. A recent study revealed that ZEB2 also influenced immune infiltration in the tumor microenvironment (57). These results demonstrated that m^7^G modification functioned in regulating biological functions.

### Comparison of Specific Immune Infiltration Landscape Among Three Subgroups

**Figure 4:**
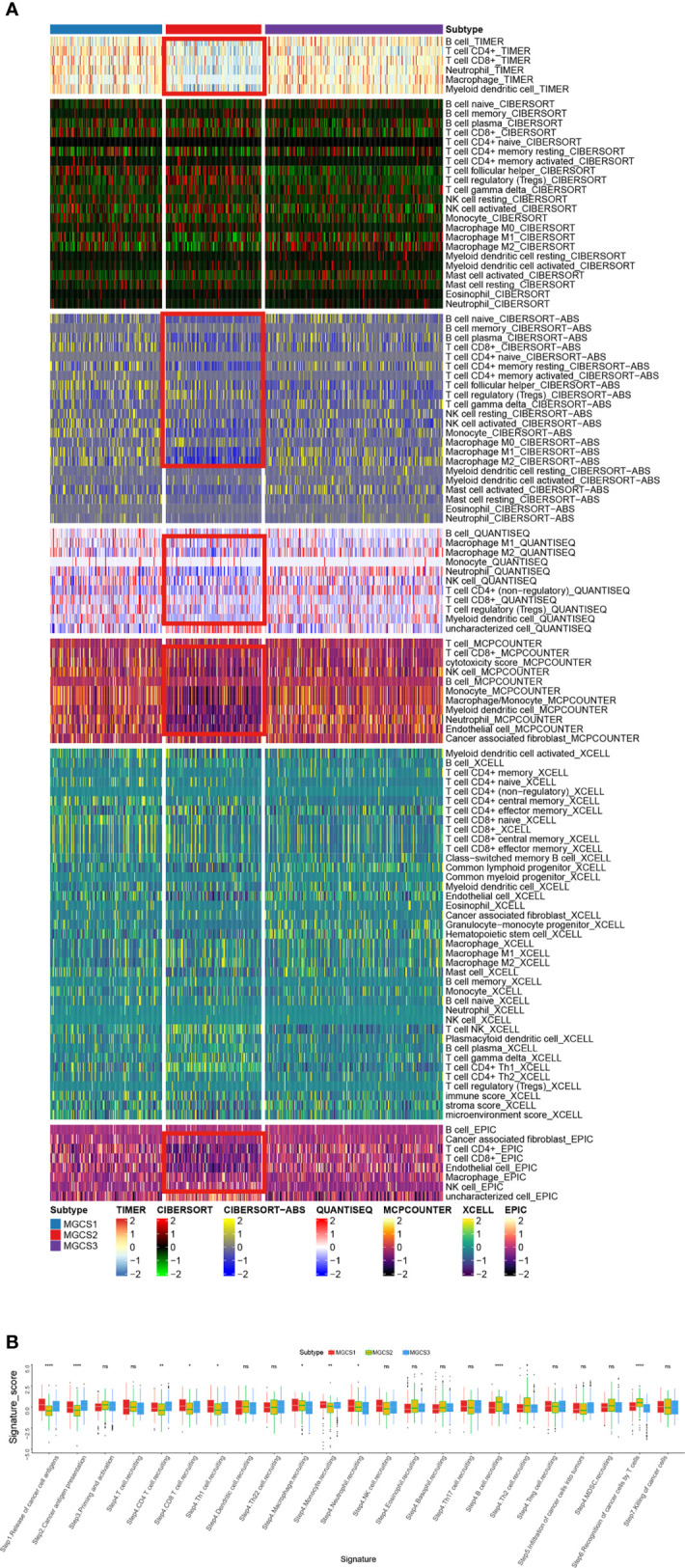
Identification of immune landscapes. **(A)** Heatmap of tumor-related infiltrating immune cells based on TIMER, CIBERSORT, CIBERSORT-ABS, QUANTISEQ, MCPCOUNTER, XCELL, and EPIC algorithms in the three subtypes. **(B)** The difference in anti-cancer immune response among three subgroups. *P < 0.05; **P < 0.01; ****P < 0.0001; NS p > 0.05.

To characterize the immune status, we compared the enrichment scores of immune-related processes across the subgroups using GSVA analysis. We found downregulated trends of chemokines, chemokine receptors, immunoinhibitors, and immunostimulators in the MGCS2 group. (**Figure S4A**). We then examined the compositions of TME infiltrating-cell types among the three m^7^G modification patterns. To our surprise, the results consistently showed that MGCS2 exhibited decreased immune cell infiltration compared to MGCS1 and MGCS3 (**Figure 4A**). Therefore, we speculated that MGCS2 could be categorized as an immune-desert phenotype, marked by the suppression of immunity. Consistent with the above survival findings, patients in MGCS2 showed a matching survival disadvantage when compared with MGCS3. We next focused on anti-cancer immune response, which can be summarized into a series of stepwise events. We also noted lower activities of many steps in MGCS2, including release of cancer cell antigens (Step 1), cancer antigen presentation (Step 2), CD4 T cell, CD8 T cell, and Th1 cell recruiting (Step 4) (**Figure 4B**). In addition, stromal score and ESTI-MATE score were significantly decreased in MGCS2 (**Figure S4B**) These results indicated that distinct immune patterns correlated with m^7^G modification.

### Characteristics of Tumor Somatic Mutation and CNV of Three Subgroups

**Figure 5:**
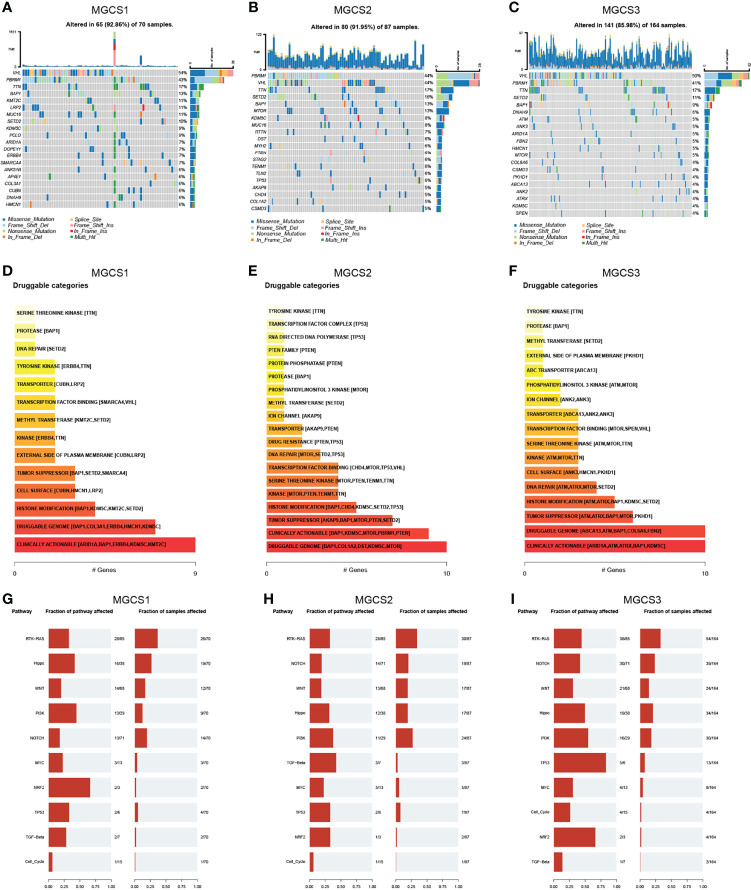
Landscapes of somatic mutation among subgroups. **(A–C)** Waterfall plot showing the mutation patterns of the top 20 most frequently mutated genes. Each column represented patients. The upper barplot showed tumor mutational burden. The mutation frequency of each gene was indicated on the right. **(D–F)** Potentially druggable gene categories from mutation datasets in MGCS1, MGCS2, and MGCS3. **(G–I)** Onco-pathway alteration frequency and the fraction of sample affected for each pathway in MGCS1, MGCS2, and MGCS3.

We then analyzed the distribution of somatic mutation differences among three groups. The top 20 frequent mutation genes are shown in **Figures 5A–C**, which indicate that the MGCS3 subtype presents a lower mutation rate than MGCS1 and MGCS2 groups. Furthermore, we investigated potential treatment targets according to the mutation data using the DGIdb database and drug interactions in maftools package. Druggable genes in three distinct m^7^G modification patterns were categorized into 14, 19, and 17 classes, respectively, including clinically actionable, druggable genome, tumor suppressor, histone modification, etc. (**Figures 5D–F**). We also evaluated the rare somatic alterations in onco-pathways (58) including RTK-RAS, Hippo, WNT, PI3K, NOTCH, MYC, NRF2, TP53, TGF-Beta, and Cell_Cycle among three groups using the R package maftools. The NRF2 and PI3K pathways were easily affected in MGCS1, while TGF-Beta and PI3K were the most affected oncogenic pathways in MGCS2. In MGCS3, TP53 and NRF2 were the most easily affected onco-pathogenic pathways (**Figures 5G–I**).

CNV differences were also compared among three clusters. MGCS2 displayed the highest rate of CNV, followed by MGCS1 and MGCS3 (**Figure S5A**). GISTIC 2.0 was used to decode the amplification and deletion regions on chromosomes of each group (**Table S3**), gain/loss percentage and GISTIC score showed similar patterns (**Figures S5B, C**). These results suggested that the distinct CNV events might result in the formation of the three subtypes.

### Drug Sensitivity Profiles of Different m^7^G Subgroups

**Figure 6:**
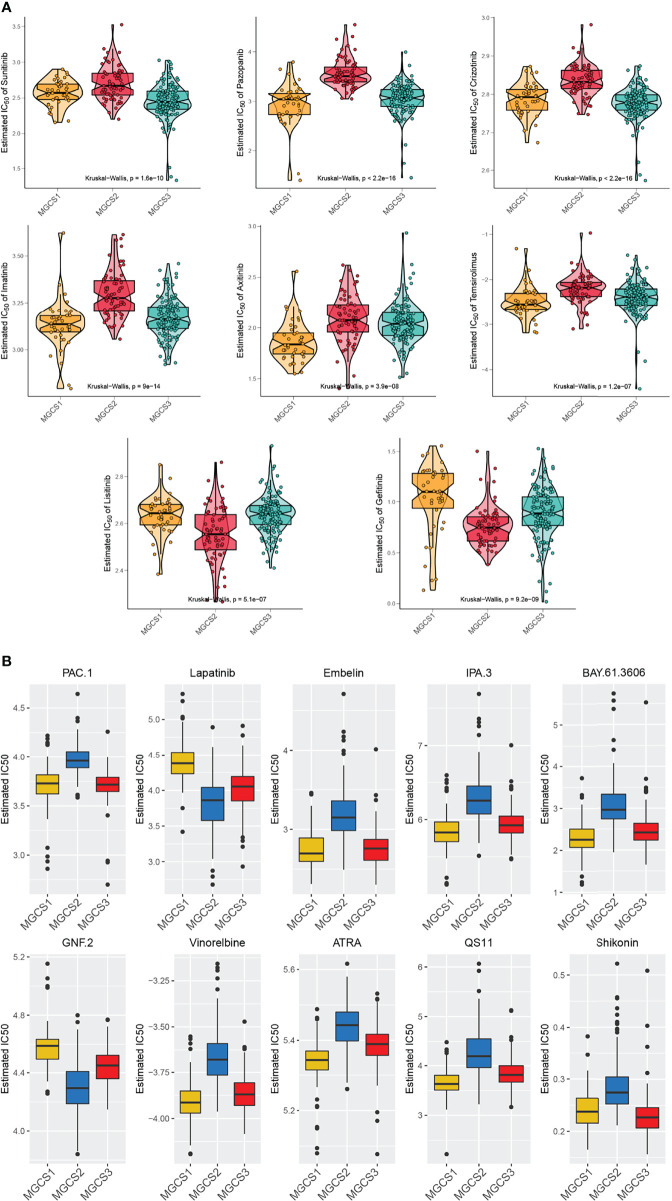
Comparison of drug sensitivity. **(A)** Estimated IC50 of the indicated molecular targeted drugs in MGCS1, MGCS2, and MGCS3. **(B)** Estimated IC50 of the potential drugs in MGCS1, MGCS2, and MGCS3.

To perform drug sensitivity analysis, the drug response data (defined by the IC50 value) were collected from the GDSC database. We found that most drugs performed worse in the MGCS2 group (**Figure 6A**), which was consistent with the previous prognosis data. Meanwhile, MGCS2 was predicted to be the more sensitive to lisitinib and gefitinib. Pazopanib, imatinib, axitinib, and temsirolimus showed a better effect on MGCS1 than other groups, while sunitinib and crizotinib demonstrated better performance in MGCS3 (**Figure 6A**). We further identified 138 small molecular drugs that could be treated as possible therapeutic approaches for ccRCC (**Table S4**). The top 10 potential drugs with the most notable differences in these groups are depicted in **Figure 6B**. MGCS1 group was sensitive to Embelin, IPA.3, BAY.61.3606, Vinorelbine, ATRA, and QS11, while the MGCS2 group had a better response to Lapatinib and GNF.2. MGCS3 group was sensitive to Shikonin. We next sought to explore the possible drugs that have an action against the oncogenic process. We evaluated the association between m^7^G regulator expression and drug sensitivity using CellMiner database. An inverse correlation was found between CYFIP1 expression and the IC50 of bendamustine, XK-469, etoposide, teniposide, valrubicin, epirubicin, and imexon (**Figure S6**), which indicated that these drugs were useful for CYFIP1 high-expressing patients. Additionally, vorinostat or nelarabine might be appropriate for SNUPN or DCP2 low-expressing patients, respectively.

### Verification of Robustness of the Subtyping Model Using External Datasets

To further evaluate the reliability of the molecular subtyping model, we used two external datasets from the GDSC renal cancer cell database and the Japan cohort for verification. For renal cancer cells, this grouping method revealed significant differences among the three clusters (**Figure S7A**). We compared the areas under the curve (AUC) of drug responses within clusters and found AUCs of GSK690693, THZ-2-102-1, TUBASTATIN A, ZM-447439, BRIVANIB, FILANESIB, GDC-0941, and SN-38 were significantly lowest in MGCS2 renal cancer cells (**Figure S7B**). Using the nearest template prediction (NTP) algorithm, subtype-specific signatures (**Table S5**) were identified from the TCGA-ccRCC, which divided the Japan cohort into three groups (**Figure S7C**). Patients with ccRCC belonging to the MGCS2 group have poorer survival than MGCS1 and MGCS3 (**Figure S7D**), in keeping with previous survival data. These results confirmed the reliability and robustness of our classification model.

### Construction and Validation of a Five m^7^G-Related Genes Risk Model

**Figure 7:**
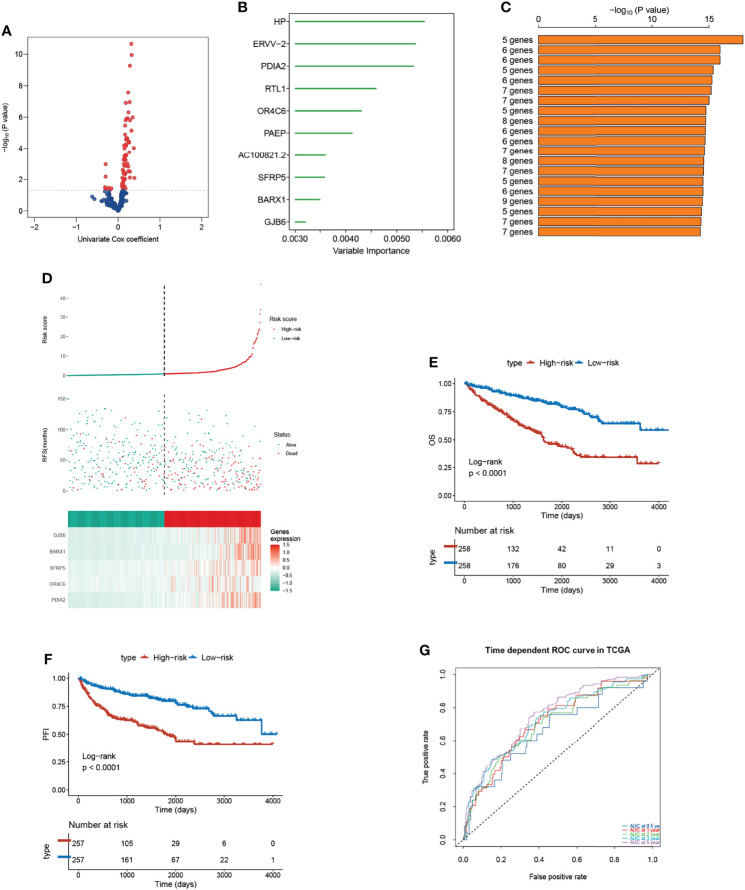
Construction of m7Sig and validation. **(A)** Volcano plot illustrating the m^7^G subtype-based genes by Univariable Cox Regression analysis. **(B)** Random survival forest analysis screening 10 genes. **(C)** After Kaplan–Meier analysis of various combinations, the top 20 signatures are ordered by the p-value. The five-gene signature was established, for it had a relatively great −log_10_(p value). **(D)** m7Sig risk score analysis of patients in TCGA-ccRCC cohort. **(E, F)** Kaplan-Meier analysis for OS (left) and PFI (right) of the high- and low-risk subtypes in TCGA-ccRCC cohort. **(G)** The time-dependent ROC curves analysis for m7Sig in TCGA-ccRCC cohort.

Since the three subtypes retained distinctive clinical outcomes and heterogeneities in biological function and immune landscape, we then utilized each subtype-based signature to construct a risk model. The Univariable Cox Regression analysis was performed to find genes that had impacts on OS (**Figure 7A**). Subsequently, 10 genes were further screened out using the random forest supervised classification algorithm (**Figure 7B**). To establish the best risk model, we used Kaplan-Meier (KM) analysis and compared the −log_10_ (*P*-value) of all risk models. Finally, the risk signature composed of five genes (PDIA2, OR4C6, SFRP5, BARX1, and GJB6) was screened (**Figure 7C**). The scoring tool (m7Sig) was constructed and the m7Sig risk score of each patient was calculated: m7Sig risk score =4.847704*PDIA2+2.849162*OR4C6+4.805007*SFRP5+6.693172*BARX1+4.046870*GJB6. To validate the risk signature applied to survival prediction, TCGA-ccRCC (**Figure 7D**) and Japan cohort (**Figure S8A**) patients were both categorized as high risk and low risk groups by using a median m7Sig score as the cut-off criterion. A comparison of the survival rate indicated that the prognosis of patients in the high-risk group was significantly worse than that in the low risk group (**Figures 7E, F**). The area under the ROC curve was used to measure the specificity and sensitivity of the m7Sig score model (**Figure 7G**). The predictive value of this m7Sig score model was also determined in the Japan cohort (**Figure S8B, C**). These results indicate that the m7Sig score could be applied to prognostic evaluation for ccRCC patients.

### Single-Cell Analysis

To determine the role of m^7^G in the TME of ccRCC, we next obtained single-cell sequence data from David’s study (27). In total, 164,722 single-cell transcriptomes from the dataset were analyzed. Then, we used t-distributed stochastic neighbor embedding (t-SNE) to classify and visualize the distribution and heterogeneity of all cells (**Figure S9A**). Notably, E1F4A1 was the most significant variously expressed gene among m^7^G genes in all cell populations of ccRCC (**Figure S9B**). These cells were also classified according to tumor stage (**Figure S9C**). We could find the expression of EIF4A1 was elevated as the tumor stage progressed (**Figure S9D**). These results indicate the potential involvement of EIF4A1 in ccRCC progression.

### EIF4A1 Expression Was Elevated in ccRCC

**Figure 8:**
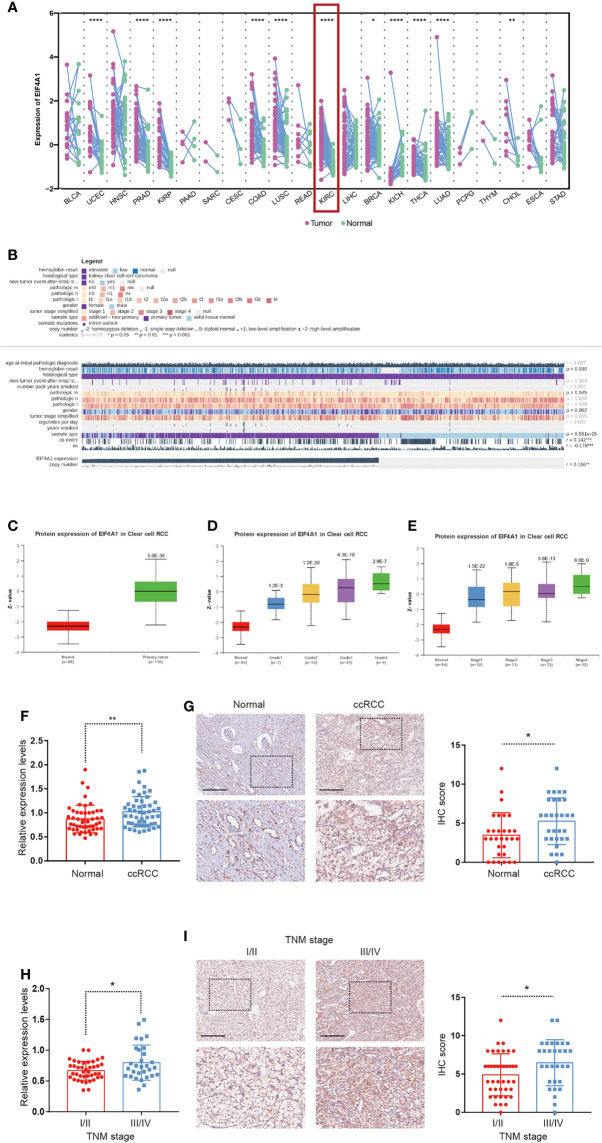
Upregulation of EIF4A1 in ccRCC. **(A)** Differential expression of EIF4A1 in paired cancer and normal tissues of 22 cancer types from TCGA database. **(B)**The correlation between EIF4A1 expression and clinicopathologic features in ccRCC from MEXPRESS database. **(C–E)** Protein levels of EIF4A1 in ccRCC samples, classified by tumor grade, and histological pathological stage using the UALCAN database. **(F, G)** RT-qPCR-determined mRNA levels and IHC staining of EIF4A1 in ccRCC and paired adjacent normal renal tissues. **(H, I)** mRNA levels and IHC staining of EIF4A1 in ccRCC samples, classified by tumor stage. Scale bar: 250 μm. *P < 0.05; **P < 0.01; ****P < 0.0001.

Given the underlying role of EIF4A1 in tumor progression, we compared the expression of EIF4A1 in tumor and paired adjacent tissues, which revealed a significantly increased level of EIF4A1 in ccRCC (**Figure 8A**). We then explored the role of EIF4A1 in ccRCC malignancy and found that EIF4A1 mutation status was associated with the immune infiltration levels of B cell, CD8+ T cell, CD4+ T cell, macrophage, neutrophil, and dendritic cell (**Figure S10**). In ccRCC, MEXPRESS-based analysis indicated that EIF4A1 levels were related to clinicopathologic features including recurrence after initial treatment, TNM classification, tumor stage, sample type, smoking history, and overall survival (**Figure 8B**). The UALCAN results indicated that EIF4A1 protein levels were upregulated in ccRCC (**Figure 8C**), which also significantly and positively correlated to cancer stages and to tumor grades of ccRCC (**Figures 8D, E**). To further verify this result, we collected and examined the levels of EIF4A1 in ccRCC and paired adjacent normal renal tissues. RT-qPCR assays and IHC staining showed that the levels of EIF4A1 were significantly higher in ccRCC tissues when compared with adjacent normal renal tissues (**Figures 8F, G**). Furthermore, EIF4A1 expression increased with the progress of the tumor stage in our cohort of ccRCC tissues (**Figures 8H, I**).

## Discussion

Clear cell renal cell carcinoma is characterized by extensive heterogeneity (59). The need for accurate diagnosis and survival prediction is urgent. There was growing evidence that showed m^7^G modification served crucial functions in embryonic stem cell self-renewal (60), tumor progression (61), and chemosensitivity (62, 63) through interaction with various m^7^G regulators. However, studies on m^7^G were not as abundant as those on other types of RNA epigenetic modifications, including m^1^A, m^6^A, and m^5^C. Furthermore, the majority of the studies focused on a single regulatory molecule. The overall characteristics mediated by the combined effect of multiple m^7^G regulators have not been fully understood.

In this study, we analyzed core genes of the m^7^G modification in pan-cancer, then identified three distinct m^7^G modification patterns (MGCS1, MGCS2, and MGCS3) in ccRCC patients. We made a comprehensive exploration of the differences among three subgroups in multiple omics dimensions. Based on the characteristic patterns of gene expression in each group, we constructed and validated a scoring tool named m7Sig, which could predict the prognosis of ccRCC patients. Given the importance of EIF4A1 through single-cell level-based analysis, we further assessed the influences of EIF4A1 on clinical features and the immune microenvironment of ccRCC.

Our data revealed high cross-correlations of multiple m^7^G regulators in pan-cancer, which indicated that there may exist a common regulatory mechanism for these genes. By the following analysis, we speculated CNV and sequence mutation may induce the abnormal expression of m^7^G genes in cancers. In our study, three m^7^G modification patterns had distinct clinical characteristics. This illustrated that dysregulated m^7^G modification affected the prognosis of patients with ccRCC. Patients in the MGCS3 subgroup had better OS and PFI relative to the other two groups, while patients in MGCS1 and MGCS2 were associated with relatively higher pathological grading and staging. Evidence is accumulating that some RNA modifications may be dynamic (64), although the characteristics of the three subgroups were completely different, pseudotime analysis also showed a risk transition trajectory.

Nowadays, ccRCC has become known as a typical representative malignancy featured by metabolic reprogramming (65, 66). In our study, enrichment analysis of the transcriptomic differences indicated that metabolic-related pathways were significantly associated with different subgroups. Metabolic processes in MGCS1 were relatively more suppressed than those in MGCS2 and MGCS3. Hence, targeting metabolic pathways could be a rationale and therapeutic opportunity. Further studies confirmed that the tumor microenvironment also displayed distinct signaling activity among the three groups. The m^6^A modification signature was significantly inhibited in MGCS2. It is widely accepted that complicated interrelations occurred between epigenetic modifications owing to the intricate interplay of epigenetic regulations (67, 68). As the most universal, abundant, and conserved modification in eukaryotic RNAs, m^6^A acts as a storm center to coordinate other epigenetic counterparts and remodel epigenetic topography (69, 70). This prompted us that epigenetics should be studied and targeted from a practical perspective. In addition, we conjectured that the functional difference among the three clusters appears to be regulated by upstream transcription factors, such as TFE3, TP53, EPAS1, and ZEB2, which requires future research. These results implied that m^7^G modification had significant implications for shaping different cellular functions.

As one of the most immunologically distinct tumor types, ccRCC exhibited the highest angiogenesis score and is frequently infiltrated with immune cells when compared with other epithelial cancer types (71). The extent of T-cell infiltration is remarkably high in ccRCC (72), which leads to marked inflammatory features. However, as the most abundant immune cells, CD8^+^ T cells display impaired anti-tumor effects, which indicates that the immune microenvironment for ccRCC is unique compared with other tumors (73). It was also reported that epigenetic modification could alter the anti-tumor immune response (74). On the basis of this, we found that these three clusters had distinct immune infiltration patterns. Most immune cells were poorly infiltrated in the MGCS2 group. Hence, MGCS2 was characterized by the suppression of immunity, equivalent to the immune-desert phenotype, also known as a cold tumor. Kim and colleagues reported that cold tumor was associated with immune escape, and impaired T-cell priming and activation (75). The process of cancer antigen presentation and T cell recruiting was also obstructed in MGCS2. It was reported that antigen presentation induced by dendritic cells in TME could initiate T cell immunity against tumors and enhance survival rates (76). This feature was in line with the observation that MGCS2 had a poorer prognosis than MGCS3. Additionally, we explored the relationship between m^7^G and CNV differences. Both copy number losses and copy number gains were higher in MGCS2, while MGCS3 showed the lowest rate of CNV. It has been reported that the extent and pattern of copy number variation were associated with cancer progression in ccRCC (77). We speculated that the likelihood of an unstable event increased with the mutational events.

As previously reported, m^7^G modification could affect the efficacy of antitumor drugs (62). We found that patients with ccRCC in different clusters exhibited distinct sensitivities to certain drugs, so our cluster models could provide more credible guidance for clinical drug use. We also investigated potential candidates for effective chemotherapy of ccRCC, particularly for patients in MGCS2 groups. Exploring the molecular mechanism behind the curative effects of these drugs promotes a deeper understanding of the pathological mechanisms.

Analytical integration of m^7^G modification patterns refined the understanding of ccRCC in tumor biology. However, considering the heterogeneity between individuals, we further incorporated the molecular features to build a risk model to predict prognosis. In this model, protein disulfide isomerase A2 (PDIA2) is a member of the disulfide isomerase family proteins. A previous study reported that PDIA2 was involved in immune infiltration and predicted immune infiltration of the colon cancer tissues (78). Olfactory receptor family 4 subfamily C member 6 (OR4C6) was reported as a possible biomarker for pancreatic carcinoma (79). However, the role of OR4C6 in ccRCC was not reported before. Secreted frizzled-related protein-5 (SFRP5) is a member of the SFRP family, which functions as a secreted antagonist by binding Wnt protein (80). Li found that SFRP5-Wnt11 signaling had profound effects on organogenesis and cancer (81). BARX homeobox 1 (BARX1), a transcription factor, is involved in craniofacial development (82) and in hepatocellular carcinoma metastasis (83). BARX1 has recently been reported for the first time to be associated with proliferation and epithelial-mesenchymal transition in ccRCC (84). GJB6 encoding Cx30 is a member of beta-connexins. It has already been shown that gap junction proteins connexins were overexpressed in the tumors when compared with normal tissue (85), which helps assemble gap junctions among adjacent cells and thus promotes gap junctional intercellular communication. In line with this, we found GJB6 correlated with poor survival of patients with ccRCC.

Our study demonstrated the aberrant gene expression pattern of EIF4A1 among a variety of cell types in TME. The level of EIF4A1 was also positively related to the ccRCC stage, which may reveal EIF4A1 as a hub gene in TME shaped by m^7^G modification. Several studies have reported the tumor−promoting effect of EIF4A1 in gastric cancer (86) and breast cancer (87, 88) by promoting oncogene translation. Our findings offer an alternate explanation that EIF4A1 could regulate m^7^G modification and influence the immune infiltration landscape in the TME.

However, there are limitations to our study. Firstly, our main findings were established through comprehensive bioinformatics analyses. Further experiment verification, including the detailed mechanism regarding how m^7^G regulators interact with each other and what downstream signaling pathways are controlled, is still needed. Secondly, although the drug sensitivities were distinct among the three subgroups, further validation experiments are required. Finally, even if we conducted verification of the prognostic model, some confounding factors, such as race and region, could not be avoided. More independent datasets are needed to reduce the potential bias.

In summary, to our best knowledge, this was the first study to explore the role of m^7^G in ccRCC and identify three m^7^G-related subtypes of ccRCC. Clinical characteristics, biological functions, immune infiltrations, genomic features, and drug responsiveness were comprehensively evaluated according to distinct m^7^G modification patterns. A robust m^7^G risk model was also constructed to predict the prognosis of patients with ccRCC. Our findings provide novel insights into the relationship between m^7^G and ccRCC, which could guide clinical decision-making.

## Data Availability Statement

The original contributions presented in the study are included in the article/**Supplementary Material**. Further inquiries can be directed to the corresponding authors.

## Ethics Statement

The studies involving human participants were reviewed and approved by Changzheng Hospital of the Naval Medical University. The patients/participants provided their written informed consent to participate in this study.

## Author Contributions

KD, DG, JS, and YB have contributed equally to this work. LW and AJ conceptualized and designed this study. ZF, YF, LQ, and WZ wrote the first draft of the manuscript. All authors contributed to the article and approved the submitted version.

## Funding

This work was supported by the National Natural Science Foundation of China (no. 81730073 and 81872074 to LW; no. 81772740 and 82173345 to LQ), Youth Project of Shanghai Municipal Health Commission (no. 20194Y0208 to JS), Foundation for Distinguished Youths of Jiangsu Province (no. BK20200006 to LQ).

## Conflict of Interest

The authors declare that the research was conducted in the absence of any commercial or financial relationships that could be construed as a potential conflict of interest.

## Publisher’s Note

All claims expressed in this article are solely those of the authors and do not necessarily represent those of their affiliated organizations, or those of the publisher, the editors and the reviewers. Any product that may be evaluated in this article, or claim that may be made by its manufacturer, is not guaranteed or endorsed by the publisher.

## Supplementary Material

Supplementary Figure 1 Workflow of this study.

Supplementary Figure 2 m^7^G regulators are dysregulated in cancers. **(A)** The expression of m^7^G regulators between normal and tumor tissues. Red represented upregulation in tumors and blue represented downregulation. **(B)** The correlation of m^7^G regulators in ccRCC expression matrix using Spearman (up-right, square) and Pearson (low-left, circle) correlation test. Red represented positive and blue represented negative correlation. **(C)** The association between expression levels of m^7^G regulators and patient outcomes. Risk-associated genes were marked with red and protective genes with blue.

Supplementary Figure 3 The risk landscape and intra-cluster heterogeneity within each subgroup. **(A)** The risk landscape of ccRCC: each point represents a patient with colors corresponding to the subtype defined previously. **(B)** The subtype of ccRCC clustered by state. **(C)** Trajectory analysis and pseudotime ordering of patients with ccRCC.

Supplementary Figure 4 Immune status among three subgroups. **(A)** Heatmap of the expression of immune-related genes among MGCS1, MGCS2, and MGCS3. **(B)** Differences in StromalScore, ImmuneScore, and ESTIMATEScore among MGCS1, MGCS2, and MGCS3.

Supplementary Figure 5 CNV alteration in three subgroups. **(A)** Barplot of genomic fractions altered in the three identified subtypes of ccRCC. **(B)** The GISTIC score of copy number profiles of ccRCC. **(C)** The gain (orange) and loss (blue) percentage of copy number profiles of ccRCC.

Supplementary Figure 6 Correlation of m^7^G regulators expression level and IC50 of different drugs obtained from CellMiner database.

Supplementary Figure 7 Verification of m^7^G-related subtypes in external datasets. **(A)** Heatmap of the expression profiles of m^7^G regulators in three subtypes of GDSC renal cancer cells database. **(B)** Drug sensitivity values for 8 compounds in the form of normalized AUC on renal cancer cell lines supplied by the GDSC database. **(C)** Heatmap of NTP in Japan cohort using subtype-specific upregulated signature identified from TCGA-ccRCC cohort. **(D)** Kaplan-Meier survival curve of the three predicted subtypes of renal cancer in Japan cohort.

Supplementary Figure 8 Verification of m7Sig in Japan cohort. **(A)** m7Sig risk score analysis of patients in Japan cohort. **(B)** Kaplan-Meier analysis for OS of the high- and low-risk subtypes in Japan cohort. **(C)** The time-dependent ROC curves analysis for m7Sig in Japan cohort.

Supplementary Figure 9 Single-cell analysis of ccRCC. **(A)** The t-SNE projections of major cell types in ccRCC. **(B)** The expression patterns of m^7^G regulators in different cell populations in TME of ccRCC. **(C)** The t-SNE plot of different cell populations according to tumor stage. **(D)** The expression patterns of m^7^G regulators in ccRCC with different tumor stages.

Supplementary Figure 10 The association between EIF4A1 mutation and immune cell infiltration in TCGA-ccRCC.

Supplementary Table 1 List of 7-Methygunaosime (m^7^G) related genes.

Supplementary Table 2 Clinicopathological features of different subtypes in ccRCC.

Supplementary Table 3 Recurrently amplified and deleted regions in the three subgroups calculated by GISTIC2.0.

Supplementary Table 4 List of discovered 138 kinds of small-molecule drugs that could be used as potential drugs for the treatment of ccRCC.

Supplementary Table 5 List of subtype-specific upregulated genes.
