# Supplementary figures and images for: Identification and Verification of m7G Modification Patterns and Characterization of Tumor Microenvironment Infiltration via Multi-Omics Analysis in Clear Cell Renal Cell Carcinoma
Source: Front Immunol. 2022 May 3;13:874792. doi: 10.3389/fimmu.2022.874792 (PMC9113293; doi:10.3389/fimmu.2022.874792)

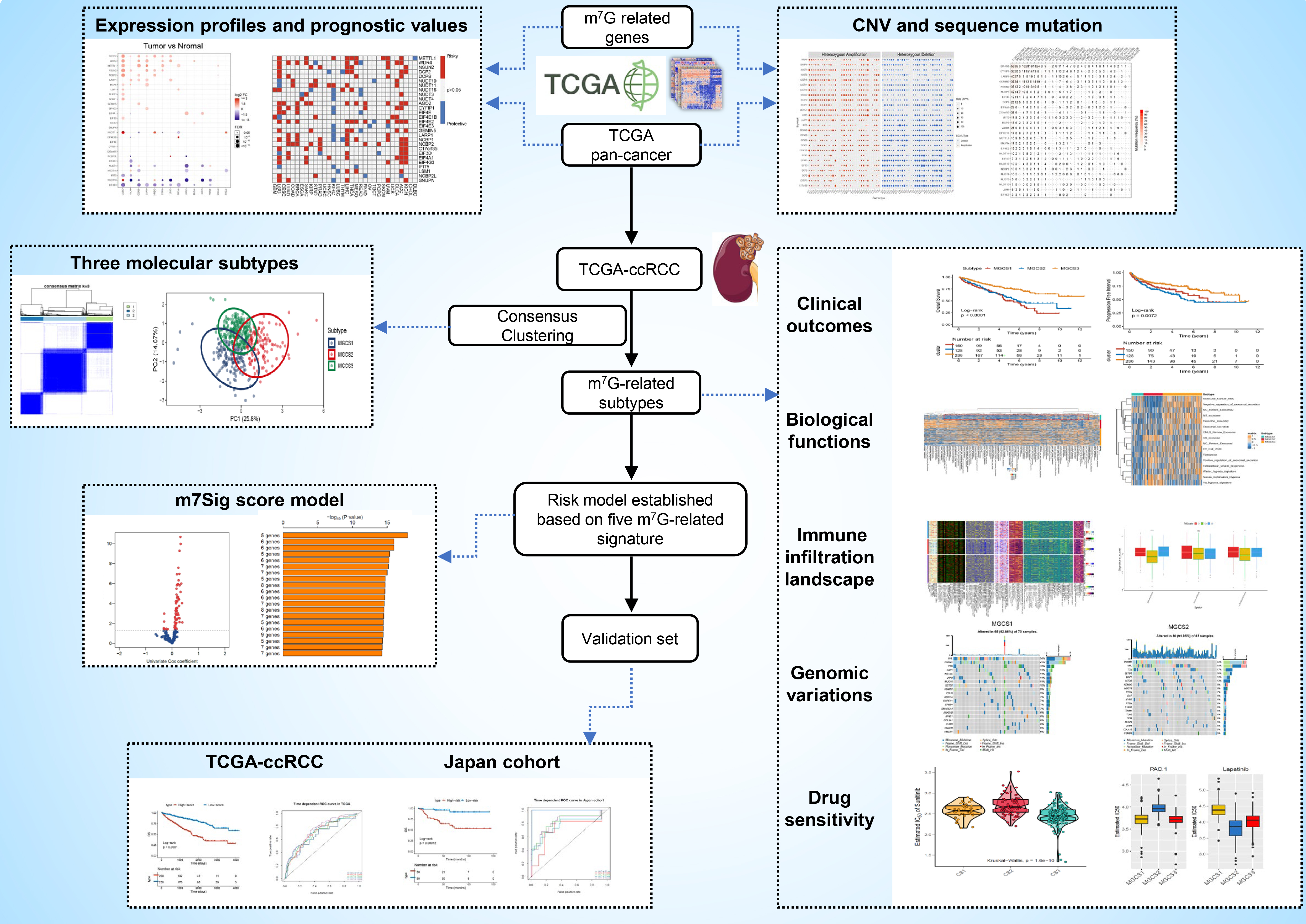

Supplement: Supplementary Figure 1 — Workflow of this study. [file Image_1.jpg]

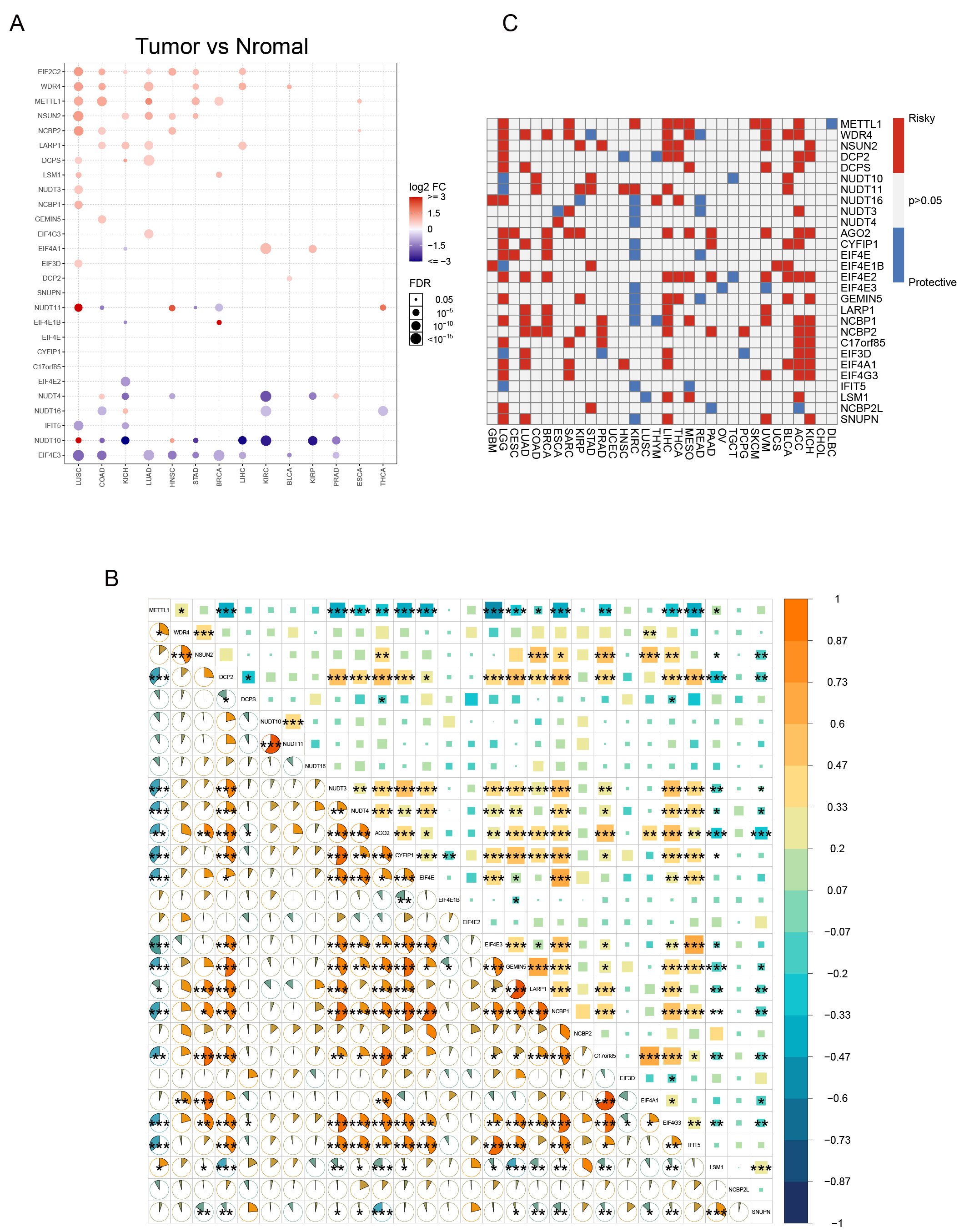

Supplement: Supplementary Figure 2 — m7G regulators are dysregulated in cancers. (A) The expression of m7G regulators between normal and tumor tissues. Red represented upregulation in tumors and blue represented downregulation. (B) The correlation of m7G regulators in ccRCC expression matrix using Spearman (up-right, square) and Pearson (low-left, circle) correlation test. Red represented positive and blue represented negative correlation. (C) The association between expression levels of m7G regulators and patient outcomes. Risk-associated genes were marked with red and protective genes with blue. [file Image_2.jpeg]

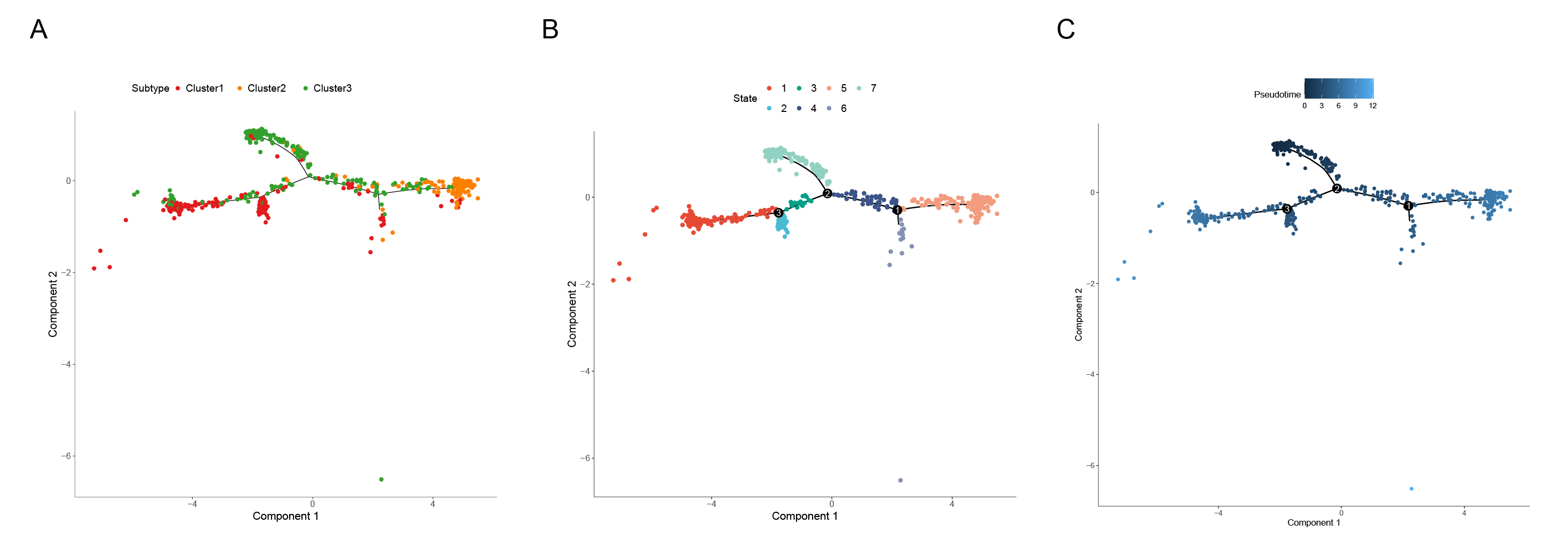

Supplement: Supplementary Figure 3 — The risk landscape and intra-cluster heterogeneity within each subgroup. (A) The risk landscape of ccRCC: each point represents a patient with colors corresponding to the subtype defined previously. (B) The subtype of ccRCC clustered by state. (C) Trajectory analysis and pseudotime ordering of patients with ccRCC. [file Image_3.jpeg]

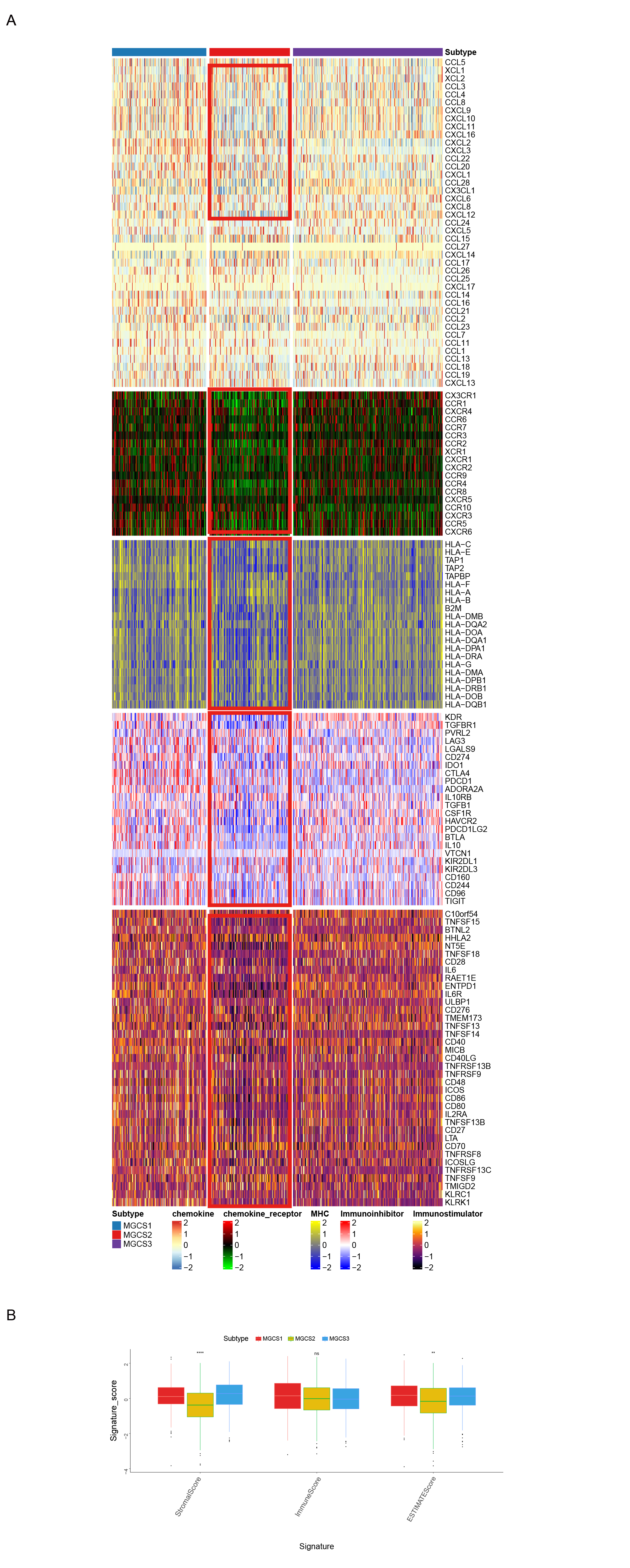

Supplement: Supplementary Figure 4 — Immune status among three subgroups. (A) Heatmap of the expression of immune-related genes among MGCS1, MGCS2, and MGCS3. (B) Differences in StromalScore, ImmuneScore, and ESTIMATEScore among MGCS1, MGCS2, and MGCS3. [file Image_4.jpeg]

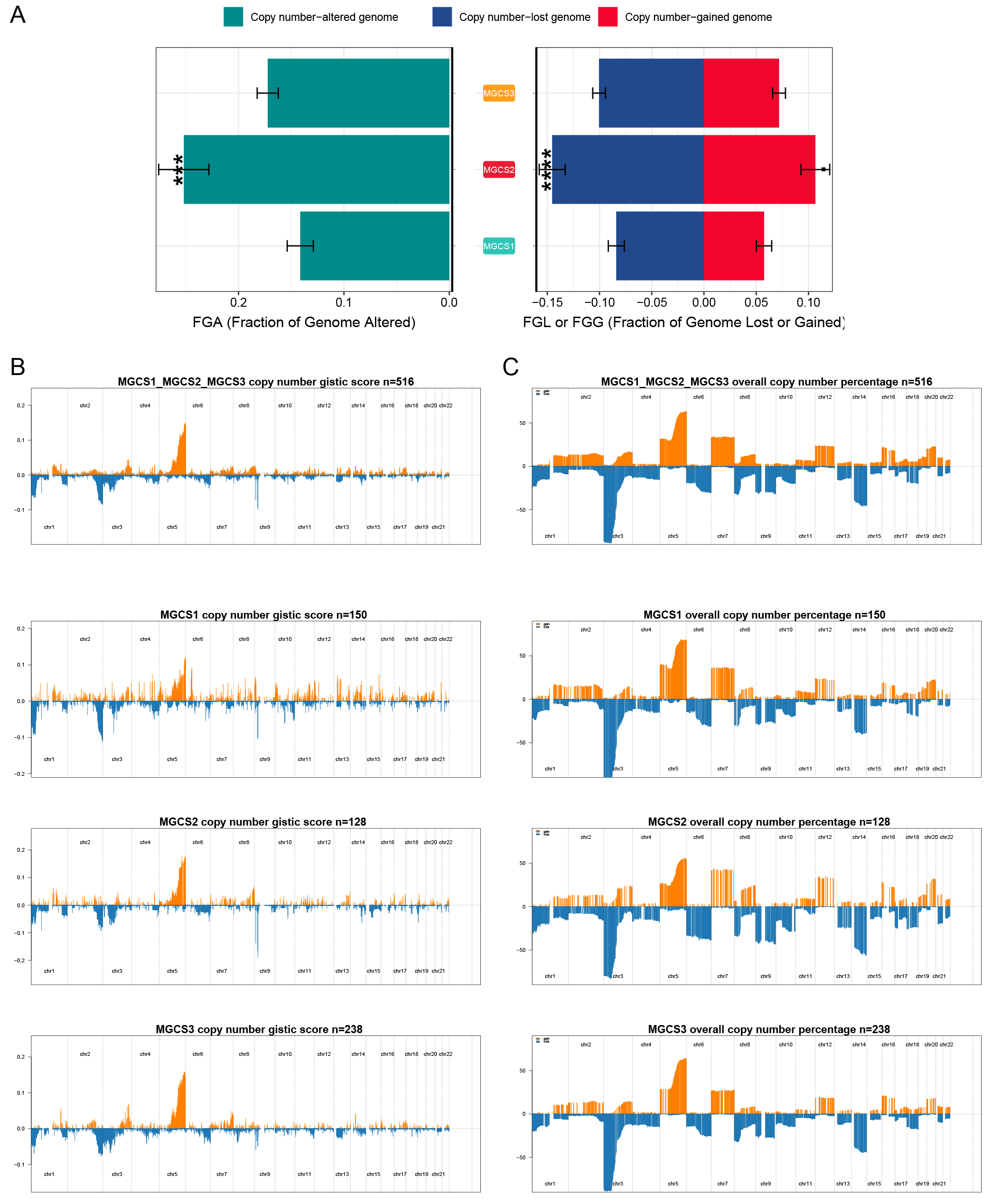

Supplement: Supplementary Figure 5 — CNV alteration in three subgroups. (A) Barplot of genomic fractions altered in the three identified subtypes of ccRCC. (B) The GISTIC score of copy number profiles of ccRCC. (C) The gain (orange) and loss (blue) percentage of copy number profiles of ccRCC. [file Image_5.jpeg]

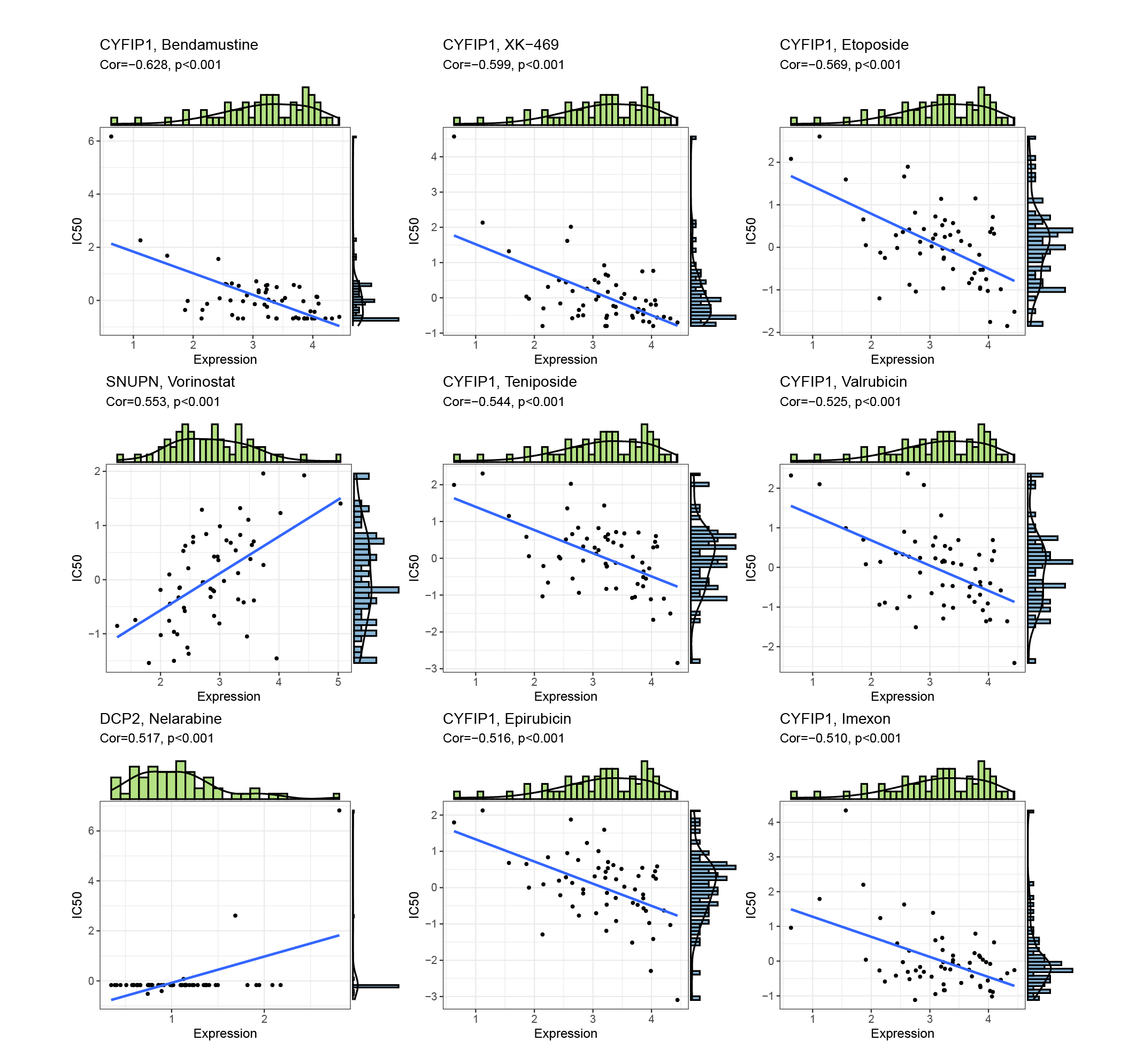

Supplement: Supplementary Figure 6 — Correlation of m7G regulators expression level and IC50 of different drugs obtained from CellMiner database. [file Image_6.jpeg]

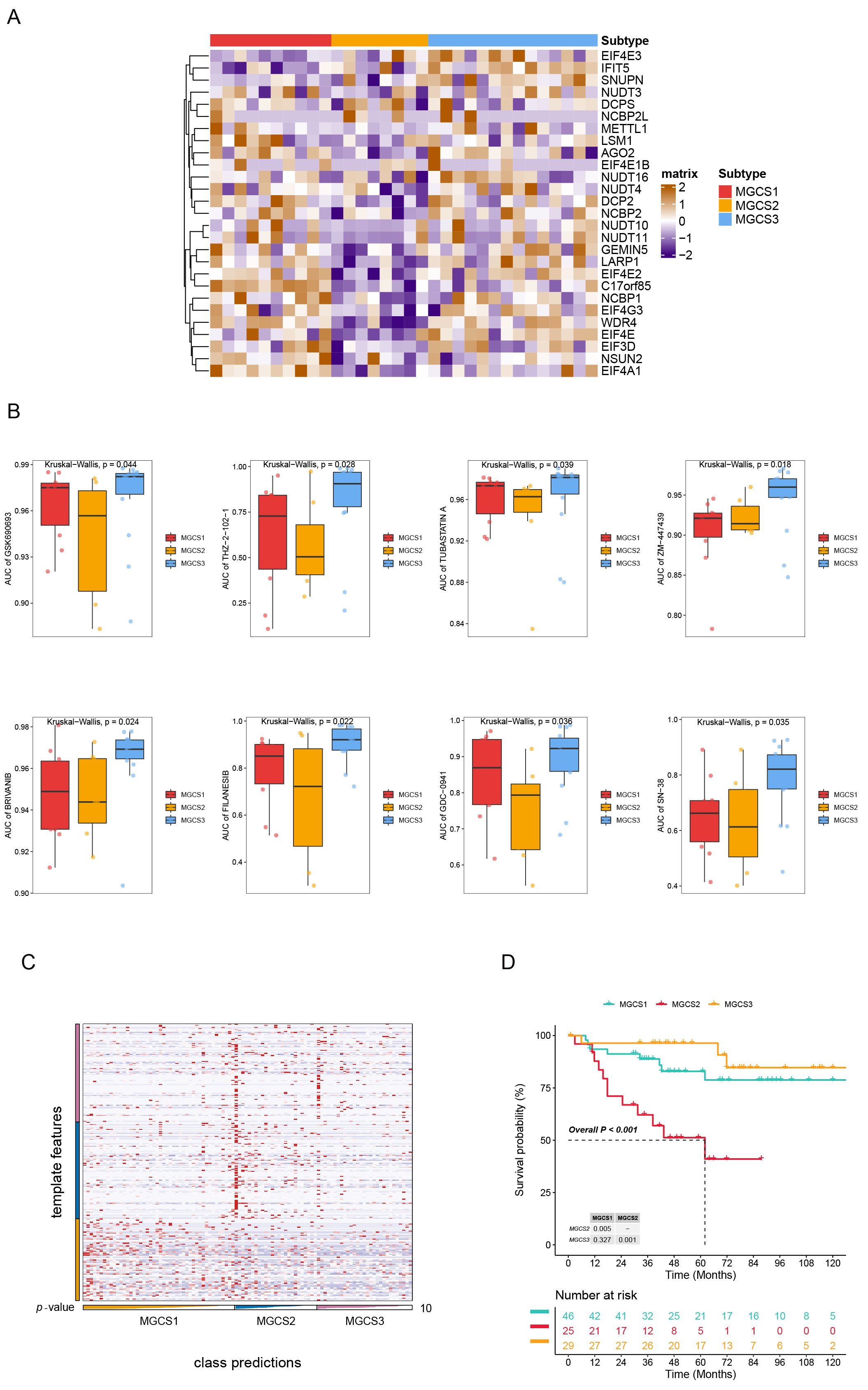

Supplement: Supplementary Figure 7 — Verification of m7G-related subtypes in external datasets. (A) Heatmap of the expression profiles of m7G regulators in three subtypes of GDSC renal cancer cells database. (B) Drug sensitivity values for 8 compounds in the form of normalized AUC on renal cancer cell lines supplied by the GDSC database. (C) Heatmap of NTP in Japan cohort using subtype-specific upregulated signature identified from TCGA-ccRCC cohort. (D) Kaplan-Meier survival curve of the three predicted subtypes of renal cancer in Japan cohort. [file Image_7.jpeg]

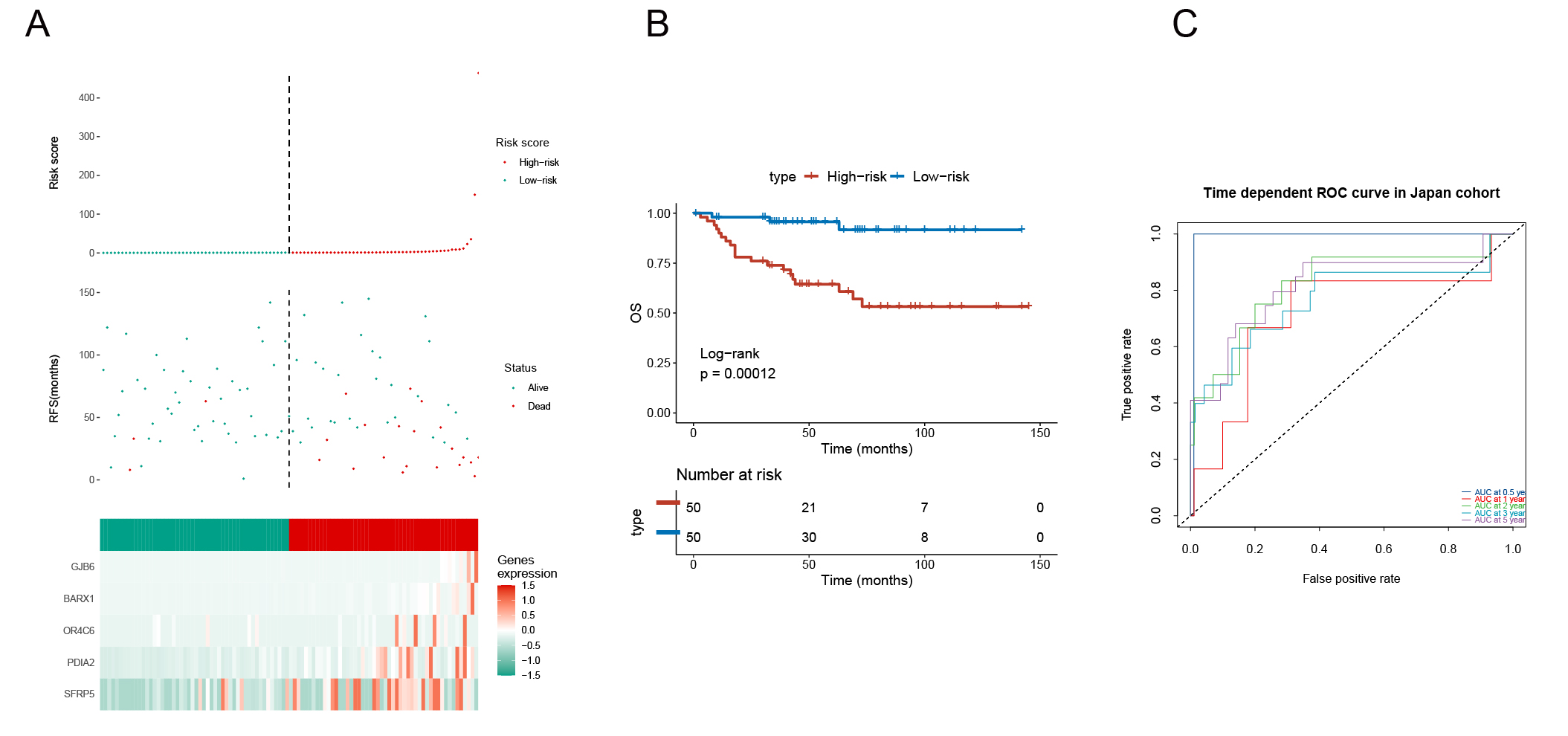

Supplement: Supplementary Figure 8 — Verification of m7Sig in Japan cohort. (A) m7Sig risk score analysis of patients in Japan cohort. (B) Kaplan-Meier analysis for OS of the high- and low-risk subtypes in Japan cohort. (C) The time-dependent ROC curves analysis for m7Sig in Japan cohort. [file Image_8.jpeg]

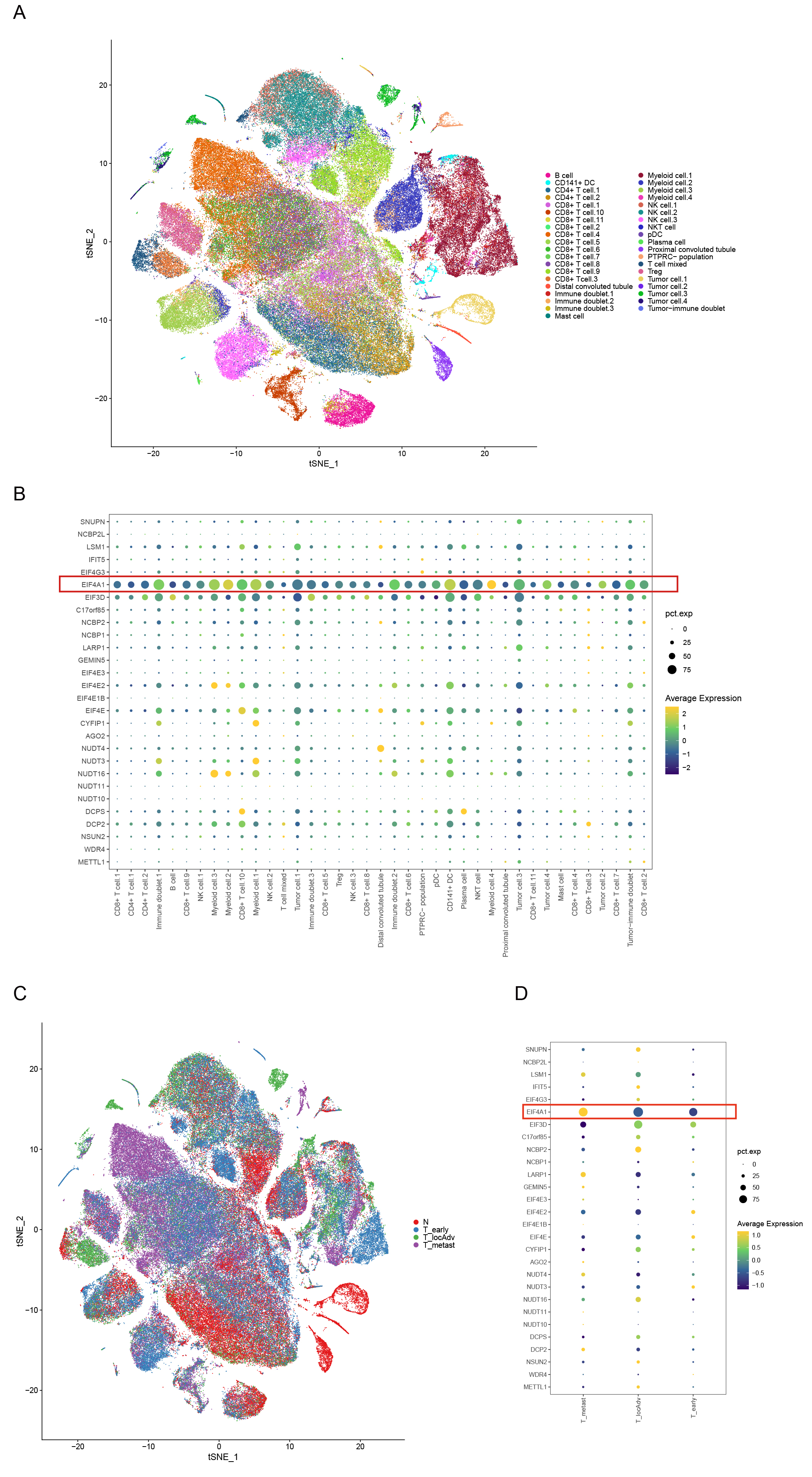

Supplement: Supplementary Figure 9 — Single-cell analysis of ccRCC. (A) The t-SNE projections of major cell types in ccRCC. (B) The expression patterns of m7G regulators in different cell populations in TME of ccRCC. (C) The t-SNE plot of different cell populations according to tumor stage. (D) The expression patterns of m7G regulators in ccRCC with different tumor stages. [file Image_9.jpeg]

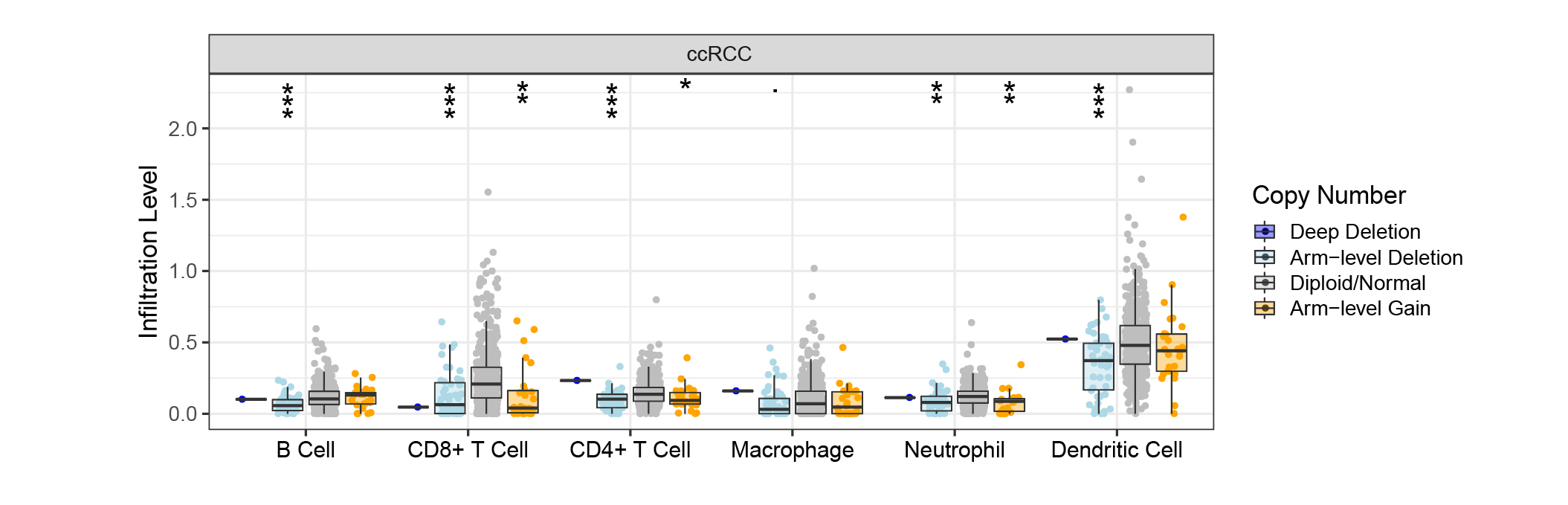

Supplement: Supplementary Figure 10 — The association between EIF4A1 mutation and immune cell infiltration in TCGA-ccRCC. [file Image_10.jpeg]
